# 3D Printed Fused Deposition Modeling (FDM) Capillaries for Chemiresistive Gas Sensors

**DOI:** 10.3390/s23156817

**Published:** 2023-07-31

**Authors:** Martin Adamek, Jiri Mlcek, Nela Skowronkova, Magdalena Zvonkova, Miroslav Jasso, Anna Adamkova, Josef Skacel, Iva Buresova, Romana Sebestikova, Martina Cernekova, Martina Buckova

**Affiliations:** 1 Department of Automation and Control Engineering, Faculty of Applied Informatics, Tomas Bata University in Zlin, Nad Stranemi 4511, 760 05 Zlin, Czech Republic; m2adamek@utb.cz; 2 Department of Microelectronics, Faculty of Electrical Engineering and Communication, Brno University of Technology, Technicka 3058/10, 616 00 Brno, Czech Republic; josef.skacel@vutbr.cz; 3 Department of Food Analysis and Chemistry, Faculty of Technology, Tomas Bata University in Zlin, Vavreckova 5669, 760 01 Zlin, Czech Republic; n_skowronkova@utb.cz (N.S.); m1_zvonkova@utb.cz (M.Z.); m_jasso@utb.cz (M.J.); buckova@utb.cz (M.B.); 4 Department of Food Technology, Faculty of Technology, Tomas Bata University in Zlin, Vavreckova 5669, 760 01 Zlin, Czech Republic; buresova@utb.cz (I.B.); r_sebestikova@utb.cz (R.S.); 5 Department of Fat, Surfactant and Cosmetics Technology, Faculty of Technology, Tomas Bata University in Zlin, Vavreckova 5669, 760 01 Zlin, Czech Republic; cernekova@utb.cz

**Keywords:** chemiresistive gas sensors, 3D printing, FDM, PLA, capillary, foods

## Abstract

This paper discusses the possible use of 3D fused deposition modeling (FDM) to fabricate capillaries for low-cost chemiresistive gas sensors that are often used in various applications. The disadvantage of these sensors is low selectivity, but 3D printed FDM capillaries have the potential to increase their selectivity. Capillaries with 1, 2 and 3 tiers with a length of 1.5 m, 3.1 m and 4.7 m were designed and manufactured. Food and goods available in the general trade network were used as samples (alcohol, seafood, chicken thigh meat, acetone-free nail polish remover and gas from a gas lighter) were also tested. The “Vodka” sample was used as a standard for determining the effect of capillary parameters on the output signal of the MiCS6814 sensor. The results show the shift of individual parts of the signal in time depending on the parameters of the capillary and the carrier air flow. A three-tier capillary was chosen for the comparison of gas samples with each other. The graphs show the differences between individual samples, not only in the height of the output signal but also in its time characteristic. The tested 3D printed FDM capillaries thus made it possible to characterize the output response by also using an inexpensive chemiresistive gas sensor in the time domain.

## 1. Introduction

Chemiresistive gas sensors are used in many applications in the form of simple detectors for more complex systems, such as electronic noses. The reason for their use is their low cost and the easily processable output signal. Their disadvantage is the low selectivity for individual chemical components in the measured gas. The material from which the sensitive resistive layer in the sensor is made can react significantly with several chemical components of the gas at the same time. For instance, the sensitive “RED” part of the sensor used in this study (MiCS-6814) can respond to up to eight different gases simultaneously [1]. The individual gases to which a given sensor may react, therefore, cannot be unambiguously distinguished from the output signal. An increase in the resolution of this method would be a useful innovation for subsequent experiments and possible practical applications. When used in simple and inexpensive applications, the gases present, to which the sensor responds, are reasonably expected. Any other gases are expected to be in negligible concentrations and thus will not affect accuracy of the measurement. As an example, the well-known measurement of ethanol in exhaled air might be used [2]. This measurement often uses an MQ-3 sensor that may respond to other substances, such as benzine, hexane, LPG, CO or CH_4_, none of which are expected during this measurement [3].

Exceptionally, unexpected gases or gases not tested by the manufacturer might be present, and an inexpensive chemiresistive sensor might respond to them. Following this, the measurements would provide inaccurate data, and, in some cases, this might lead to economic harm, e.g., in the food industry, by the discarding of satisfactory resources that were flagged as not satisfactory by, for example, an electronic nose [4]. Other cases of misdetection might cause false alarms with another economic or delaying effect on the monitored process.

One way to increase the resolution of chemiresistive gas sensors is to modify the experimental setup by connecting to another device to provide preparative separation of the sample gases. In this study, we evaluate the possibility of using 3D FDM (fused deposition modeling) printing to produce a 3D column attached to chemiresistive gas sensors to increase the accuracy of detection of individual sample gases.

The main approaches in 3D printing include material extrusion (which includes FDM), stereolithography (SLA) or photopolymerization in vats (tanks), powder bed melting, material blasting, binder blasting and plate lamination or the production of laminated objects [5]. FDM printing is the world’s most widespread 3D printing method, offering a very good ratio between the acquisition costs (equipment, material, energy consumption, equipment repair costs) and the resulting quality of the printed object. A higher quality printed object could be achieved using another technology, such as SLA, but with significantly higher acquisition costs and material costs [6,7].

In recent years, various 3D printing methods have gained significant popularity among the scientific community and not only in the field of chemical analysis, where 3D printing is mainly used to produce a wide range of instruments for processing and subsequent sample analysis [5,8]. The high value of 3D printing lies primarily in liquid chromatography methods, where it represents the possibility of producing a column with a complex internal geometry that affects the efficiency of chromatographic separation [9]. The production of chromatographic columns with defined morphologies for very high separation efficiency is becoming a very interesting approach in the field of liquid chromatography, along with the increasing affordability, speed and material flexibility of 3D printing [10].

The application of 3D printing in chromatographic methods is not only promising for liquid chromatography, but studies are also looking at printing columns for gas chromatography, e.g., printing metallic GC columns for the separation of alkene mixtures [11] or printing miniature high-resolution SLA columns for a compact and mobile gas sensor-based GC system [12].

The significant potential of 3D printing also lies in the biomedical field [13] or for food printing applications [14,15]. However, beyond the aforementioned scientific field, 3D printing technology also offers practical solutions in the manufacturing of different types of sensors and biosensors [16], and 3D printing also holds the potential to improve the performance of these devices [17]. Thus, the use of 3D printing represents a new and interesting approach in the field of gas sensors [18].

### 1.1. Scientific Hypotheses

**Hypothesis:**  *The preparative separation of the gas mixture sample using a 3D printed FDM capillary can modify the sample so that it can be characterized in the time domain of the output response using an inexpensive chemiresistive sensor.*

### 1.2. The Target

The aim of the study is to design, fabricate and test a 3D printed capillary, fabricated using the FDM method, for preparative separation of a gas sample whose concentration is measured by a selected chemiresistive sensor. According to the obtained initial response, the sample can be characterized not only in terms of the magnitude of the output signal but also in terms of its changes over time (chromatographic determination). The measurements are mainly focused on the evaluation of food samples.

## 2. Materials and Methods

### 2.1. D printed Capillary

#### 2.1.1. Model Creation

**Figure 1:**
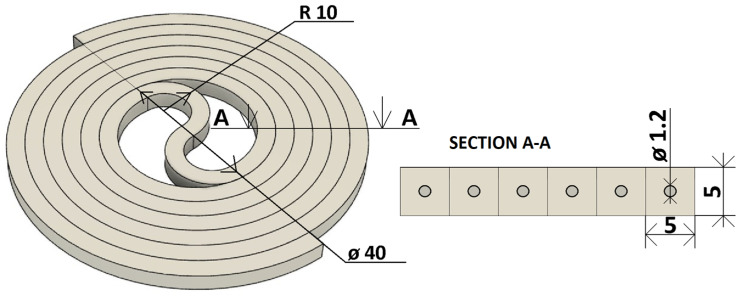
Basic model of a 3D printed capillary.

**Figure 2:**
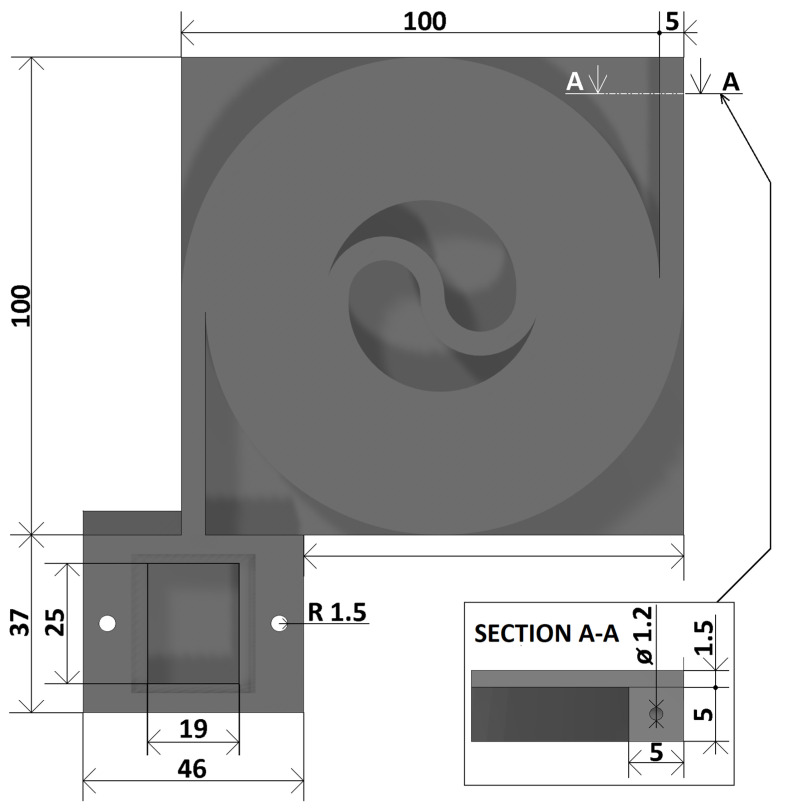
3D printed model of a single-tier capillary with measuring chamber for gas sensor and cap.

In the first stage of the study, a basic model of the capillary arrangement in a single tier was proposed. The basic arrangement was inspired by the papers by Sandron et al. (2014) and Lucklum (2015) [12,19]. In the design program Fusion 360 (Autodesk, Inc., San Rafael, CA, USA), 2 coils with a square cross-section of 5 mm × 5 mm were created in a straight helical layout with the diameter of the inner threaded circle set to 40 mm. The number of turns was chosen to be 3 with a 10 mm pitch from one edge of the turns to the other. The turns were then inserted into each other and connected by an S-shaped section of the same cross section as the turns. In this way, the object shown in Figure 1 was created and, in later design stages, formed the basis of a 105 mm × 100 mm capillary model. Furthermore, a support plate was added to the resulting model to reinforce the object and prevent damage, and a 50 mm long tube was added to serve as the inlet and outlet sections. The complete model of the single-tier capillary with the gas sensor measuring chamber and cap is shown in Figure 2.

**Figure 3:**
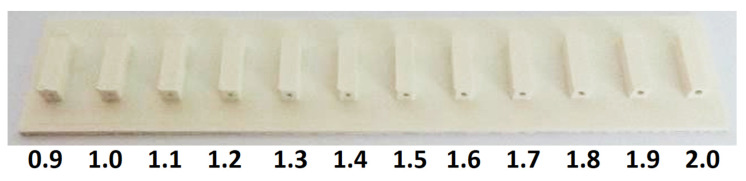
Sample from the optimal capillary diameter test (diameter in mm).

In the first model produced with a capillary diameter of 0.9 mm, the capillary was sinking and clogging. For this reason, a simple test was carried out to produce capillaries with different diameters. The test resulted in an optimal capillary diameter of D = 1.2 mm, which was used in other parts of the study. A sample from this test is shown in Figure 3.

The basic model of a single-tier capillary made from PLA (lactic acid polymer) was further stacked in the form of tiers. In this way, two more models with 2 and 3 tiers of capillaries were created. Using a model with more tiers allows longer capillaries with bigger inner surface to be printed, having direct effect on separation efficiency of the gas sample. Longer capillaries with bigger inner surfaces provide better separation efficiency at the expense of prolonging the analyses and their price. Today, commercially available capillaries for gas chromatography are available in lengths from 10 to 60 m. The length of printed three-tier capillary was calculated to be 4.7 m (Table 1) and thus may be comparable to them [20,21]. The connection between the stories was provided by a semicircular capillary with a diameter of 100 mm and a total pitch of 5 mm (inclination less than 3°). The last model was a measuring chamber for the gas sensor only with a cap and a 30 mm long tube. In this model, the gas sample will be fed directly to the sensor without any previous modifications. The actual volume of the measuring chamber without the inserted sensor is 1.57 mL (25 mm × 19 mm × 3.3 mm). Lengths, volumes, weights and inner surfaces of the capillaries are given in Table 1.

#### 2.1.2. Capillary Realization

After designing the individual objects, the designs were exported to .stl format files and transferred to the Flashprint program (Zhejiang Flashforge 3D Technology Co., Ltd., Jinhua, China), enabling the preparation for 3D printing of the design on a Flashforge Dreamer FDM printer (Zhejiang Flashforge 3D Technology Co., Ltd., Jinhua, China). The size of the printing platform for this printer is 230 mm × 150 mm × 140 mm. This size and other parameters of printer had to be taken into account when designing the capillaries.

**Figure 4:**
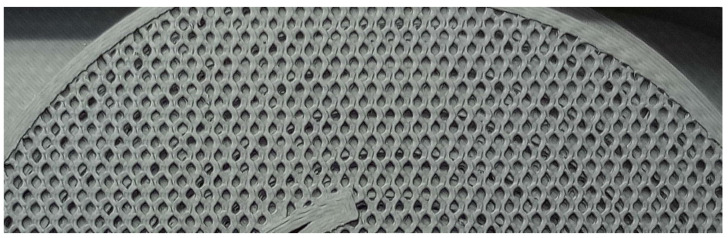
Two-tier capillary filling.

To save material, the thickness of the layers was set to 0.2 mm for 3-tier capillaries. For the other designs, the layer thickness was chosen to be 0.1 mm. To save material, full fill was not chosen in appropriate places, only the “honeycomb” type. The details are shown in Figure 4.

FilamentPM’s PLA (lactic acid polymer) bioplastic (Prusa Polymers a.s., Prague, Czech Republic) was used as the base printing material. It is an easy-to-print, low-cost material with good hardness and stiffness and a low tendency to deform during printing. However, the disadvantages are low temperature resistance, poorer workability, low resistance to liquid chemicals and also poorer adhesion of layers during printing (Data Sheet Prusament PLA) [22]. Given the expected use of gas samples and air as carrier gas, the chemical resistance of PLA material is considered sufficient.

**Figure 5:**
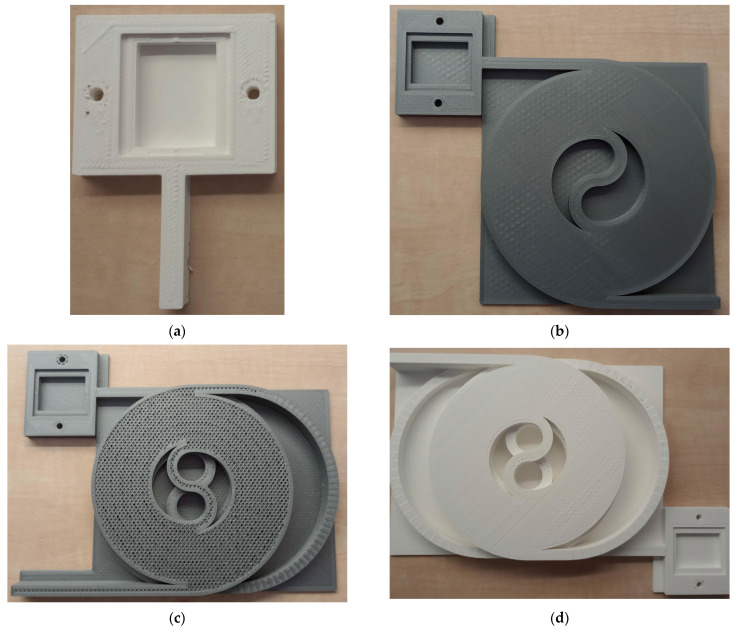
3D printed objects implemented in the study (**a**) measuring chamber without capillary; (**b**) single-tier capillary; (**c**) double-tier capillary; (**d**) triple-tier capillary.

All realized capillaries are shown in Figure 5. These are

- Measuring chamber without capillary (hereafter denoted 0L);
- Single-tier capillary (hereafter denoted 1L);
- Two-tier capillary (hereafter referred to as 2L);
- Three-tier capillary (hereafter denoted 3L).

### 2.2. Detection

MiCS 6814 sensor (SGX Sensortech, Neuchâtel, Switzerland) was selected as the basis for the sensor system. The sensor is used to measure pollution from automobile exhausts and to detect agricultural and industrial odors. The system is “a compact MOS sensor with three fully independent sensing elements on one package” (Data Sheet MiCS-6814 1143 rev 8) [1]. Individual independently operating sensors respond to groups of gases designated as

- Reduction (RED sensor, further referred to as CO sensor)—reacts mainly to carbon monoxide CO, but also to ethanol, hydrogen, ammonia, propane, methane, iso-butane or H_2_S,
- Oxidation (OX sensor, further referred as NO_2_ sensor)—provides signal mainly in the presence of nitrogen dioxide as well as NO or hydrogen gas,
- NH_3_ (NH_3_ sensor)—reacts mainly to ammonia NH_3_, but also to ethanol, hydrogen, propane or iso-butane.

MiCS 6814 might further partially detect other compounds (e.g., bigger, complex molecules) or different concentrations on which the sensor might not have been tested by the manufacturer (e.g., due to rapid sensor destruction).

**Figure 6:**
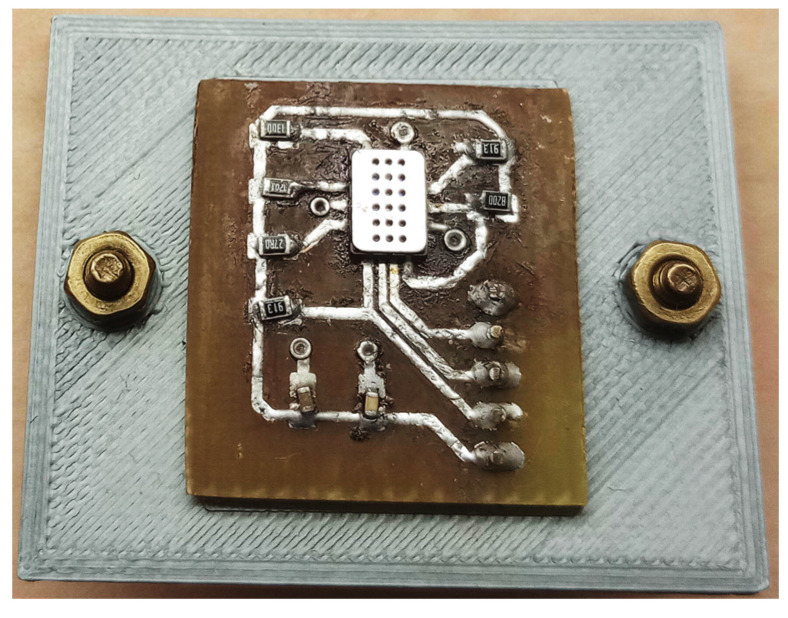
Printed circuit board with sensor and cap (state after testing).

The basic sensor electrical circuit was wired according to the manufacturer’s recommendation with total voltage equaling +5 V. The individual “sensor chips” are connected via 91 kΩ resistors for the NH_3_ and CO sensor chips and 1.2 kΩ for the NO_2_ sensor. In addition, 100 nF and 1 µF capacitors are connected to the power supply. The printed circuit board with the sensor and cap is shown in Figure 6.

**Figure 7:**
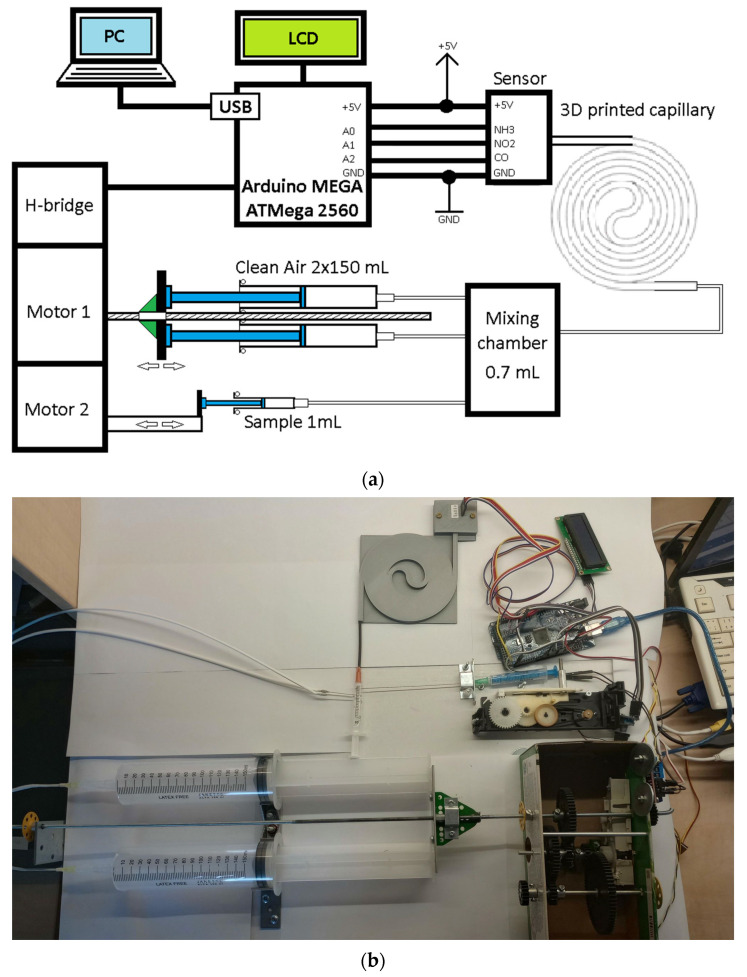
Experimental measuring system (**a**) block diagram; (**b**) photograph.

Experimental measurement system is based on Arduino MEGA 2560 system, controlled by ATmega 2560 microcontroller (Microchip Technology Inc., Chandler, AZ, USA). The microcontroller senses the signals from the sensor and transmits them via USB port to a PC-class computer (sampling rate is 1 sample/s during measurement). Display shows instructions for device operation. The microcontroller also controls two DC motors via H-bridge with L298N circuit. The first motor operates the delivery of clean air, serving as a carrier gas, from two 150 mL syringes into a 0.7 mL mixing chamber. The motor is powered directly from a separate stabilized +5 V supply and has been mechanically connected to a gearbox designed and built specifically for this device. This ensures that clean air is pumped into the mixing chamber at a rate of approximately 18.6 mL/min. Half the pumping rate can be used if one of the syringes is disabled. A second motor controls the dispensing of a 1 mL sample from a 2 mL syringe. Block diagram and photograph of the experimental measurement system are shown in Figure 7.

The measurement starts by washing the 3D printed capillary and mixing chamber with clean air. At time t = 60 s, the sample of volume 1 mL is injected into the mixing chamber followed by pumping of clean air for at least 600 s (usually 900 s). When the measurement is complete, the syringes are flushed with clean air and the entire system is washed 4 times with 150 mL of clean air.

### 2.3. Samples

Food products available in the regular food chain were used as the baseline samples for the measurements. Neither food products nor gas samples derived from them were modified in any way before the analysis. Prior to analysis, a 1 mL sample of the odor was drawn into the syringe directly above the sample and inserted into the measuring system.

The primary food sample selected was “Vodka” (GAS Familia, s.r.o., Stará Ľubovňa, Slovakia) with an alcohol content of 37.5% vol. The manufacturer describes the product as being composed only of very fine refined spirit of high quality and demineralized water (expected composition is 62.5% vol. water and 37.5% vol. ethanol). Simple composition of the beverage allows for the sensor’s response to ethanol, specifically, with absence of any side signals to be detected. Following this, “Vodka” presents itself as an appropriate standard for the study and thus was chosen as a standard for ethanol and water vapor.

The next alcoholic beverage sample tested was “Tuzemský” (GAS Familia, s.r.o., Stará Ľubovňa, Slovakia) with an alcohol content of 37.5% vol. The manufacturer states the composition as ethanol, aroma, sugar, coloring and ammonia caramel. This sample is thus equal to the “Vodka” in terms of ethanol content but consists of more components to which the sensor can respond. Further measuring was aimed at the possibility of determining ammonia in spoiled food. For this reason, seafood and skinless chicken thigh meat purchased from a supermarket chain the day before the expiry date were used. The samples were placed in a sealed 50 mL container and then left at room temperature for 1 day for the seafood sample and 3 days for the chicken sample. The cap was pierced with a needle to obtain a sample odor. Seafood and chicken meat are food categories prone to microbial spoilage, and a simple chromatographic system might help to detect spoilage products in an inexpensive way [23,24,25]. The last two samples were selected due to the easy availability of various other gases. The first sample was the gas from a Royce cigarette lighter, commonly reported as butane (97.5%) and propane (1.2%) (Safety Data sheet Royce Gas for lighters) [26]. The second sample was EBELIN acetone-free nail polish remover, 125 mL, for sensitive nails, with aloe vera fragrance (dm-drogerie markt, Karlsruhe, Germany), with ethyl acetate, alcohol denat., aqua, prunus amygdalus dulcis oil parfum and benzyl alcohol.

Alcohol, seafood and other samples were chosen for the study due to the fact that all of them produce mixtures of substances to which MiCS-6814 subunits are sensitive. Following this, it was suitable to pre-process the mixture using the capillary mentioned above, as this allowed for more precise following data assessment. Another reason behind choosing the samples was their availability and their common usage in households (handling safety).

All samples were measured in triplicate, except for the “Tuzemský” alcoholic beverage sample, for the normal airflow measurements of the three-tier capillary, and the nail polish remover sample, which was at risk of sticking and damaging the capillary walls.

The measurement results were described by the digital value d [-], which represents the sensor’s output voltage in the range of U = (0–5) V, connected to the microcontroller input pin and converted to the range of values d = (0–1023) using an A/D converter.

All data obtained from the measurements were processed and evaluated using Microsoft Excel 2019 (Microsoft Corporation, Redmond, WA, USA). From the individual curves obtained, the mean resulting curve was calculated and smoothened using a moving average (m = 11).

Then the signal was normalized to 30 s from the start of the measurement, the signal for clean air was subtracted from the measured signal and the result was converted to % according to this equation:

data = ((d_s_/d_s30_) − (d_air_/d_air30_)) × (−100) (1)

where

d_s_ is the value for the measured gas at a given time,

d_s30_ is the value for the measured gas at 30 s,

d_air_ is the value for air at a given time,

d_air30_ is the value for air at 30 s.

The absolute increment was next calculated and smoothed using the moving average (m = 11).

## 3. Results and Discussion

### 3.1. Capillary Parameters Influence

**Figure 8:**
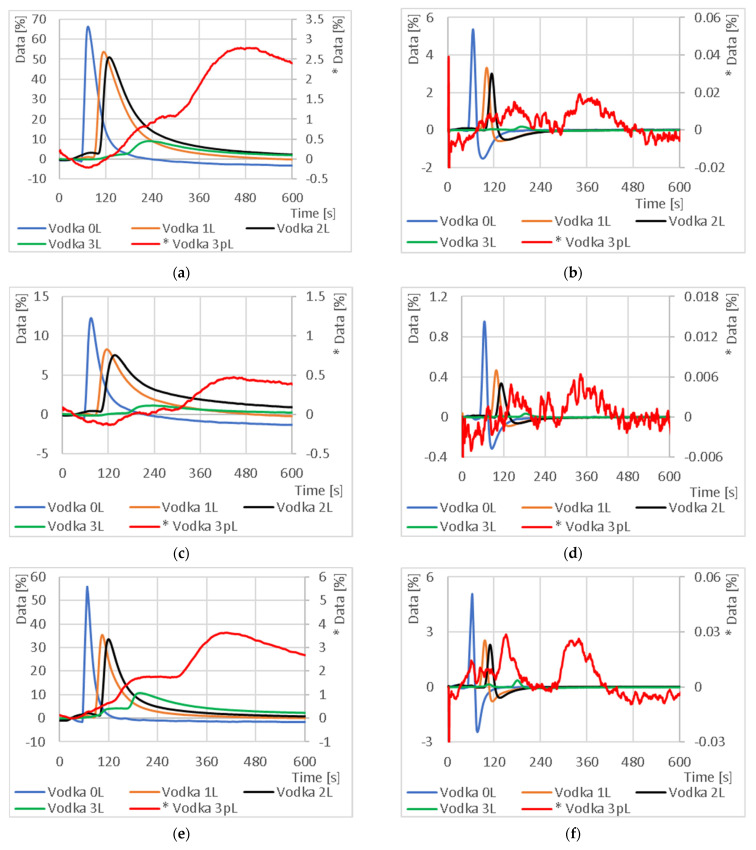
Comparison of output response and absolute increments for different 3D printed capillary arrangements using a “Vodka” sample: (**a**) output responses from the NH_3_ sensor; (**b**) absolute increment from the NH_3_ sensor; (**c**) output responses from the NO_2_ sensor; (**d**) absolute increment from the NO_2_ sensor; (**e**) output responses from the CO sensor; (**f**) absolute increment from the CO sensor. The “* Vodka 3pL” signal has a separate axis (right) on all plots due to the small signal level.

In the first phase, the influence of capillary parameters on the measurement results was investigated. The test was performed on a selected standard sample, “Vodka”. The results are shown in Figure 8. The numbers for each curve indicate the number of capillary tiers (1L, 2L and 3L). The code “0L” indicates a direct measurement of the sample with no capillary, and the “p” in the code indicates a measurement with half the air flow. This coding may also be used for other samples.

Graphs comparing the output response and absolute increment for different 3D printed capillary arrangements using a sample of vodka (37.5% ethanol) illustrate the shift in peak signal response as a function of capillary length for a normal carrier gas flow rate. As the capillary length increases, the peak also decreases. Further “stretching” of the peak can be accomplished by reducing the air flow rate from the normal flow rate to half the flow rate.

A study by Phyo et al. [11] confirmed the suitability of the 3D printing method as a new approach for potential mass column production. A spiral column of Ti6Al4V powder grade 23 with a length of 1 m was printed on a planar plate. The resulting column dimensions due to the helical arrangement were 34 mm × 33 mm × 2 mm, with an inner diameter of 500 µm. After subsequent deposition of the stationary phase, OV-1, this column was able to partition a mixture of alkanes, aromatic hydrocarbons, alcohols and ketones with sufficient peak resolution (R > 1) within two to three minutes. Subsequently, in another experiment, the column successfully separated a gaseous mixture of twelve alkanes: C9–C18, C22 and C24 [11]. Based on this research, it is possible to further reduce the column diameter and increase the print accuracy using the FDM method (e.g., using a nozzle with a diameter of 0.2 or 0.1 mm, reducing the height of one layer down to 0.03 mm depending on the 3D printer used and further optimizing the FDM printing parameters), as the internal capillary treatment can fix the stationary phase with the possibility of separating different organic compounds (presuming the inertness of the printed column is preserved).

As shown in Figure 8, the highest signal shift in time was achieved using a three-tier column with an internal area of 176.96 cm^2^. The results further indicate that the mixture separates during the measurement. This phenomenon was observed for capillary 3L and half the flow rate of the carrier gas, where, for example, in Figure 8f, there are two peaks—a peak at 150 s for substance A and a peak at 335 s for substance B. Identification of the individual peaks and specification of their retention time is a matter for further research. However, the disadvantage of this configuration is low sensitivity. Based on these measurements, it is possible to compare single-component standards with food samples in the future based on time elution, to characterize not only the signal intensity but also the signal increment over time and to identify which components are present in the food.

The usefulness of using clean air as a carrier during gas chromatography for 3D printed columns is consistent with the study by Zaidi et al. [27], in which the ability to detect ethylene at a concentration as low as 2.3 ppb using a printed column was verified by high-resolution stereolithography using an electrochemical gas sensor. In the column used, the separation of ethylene gas and water vapor also occurred [27]. The use of clean air as a carrier within the chromatography set-up helps the overall simplicity of the system, since there is no need for special equipment associated with carrier gas storage; therefore, it is advisable to develop this trend in the future.

The separation and detection of ethylene is also addressed in the study by Lucklum et al. [12], who compared 3D printed chromatographic columns with a total length of 36 cm (in the case of a single helix) and 45 cm (in the case of a compound helix). The use of a stacked column with a total length of 45 cm showed better separation properties than a column with a total length of 36 cm. The results of the study also suggest that the higher packing compactness of the stationary phase in the circular cross-section and the GC column material play a role in increasing the separation efficiency on the column [12]. It is critical, therefore, to further investigate the design of the printed columns themselves in future experiments and to observe how the used parameters affect the separation efficiency.

### 3.2. Capillary Applications in the Food Industry

**Figure 9:**
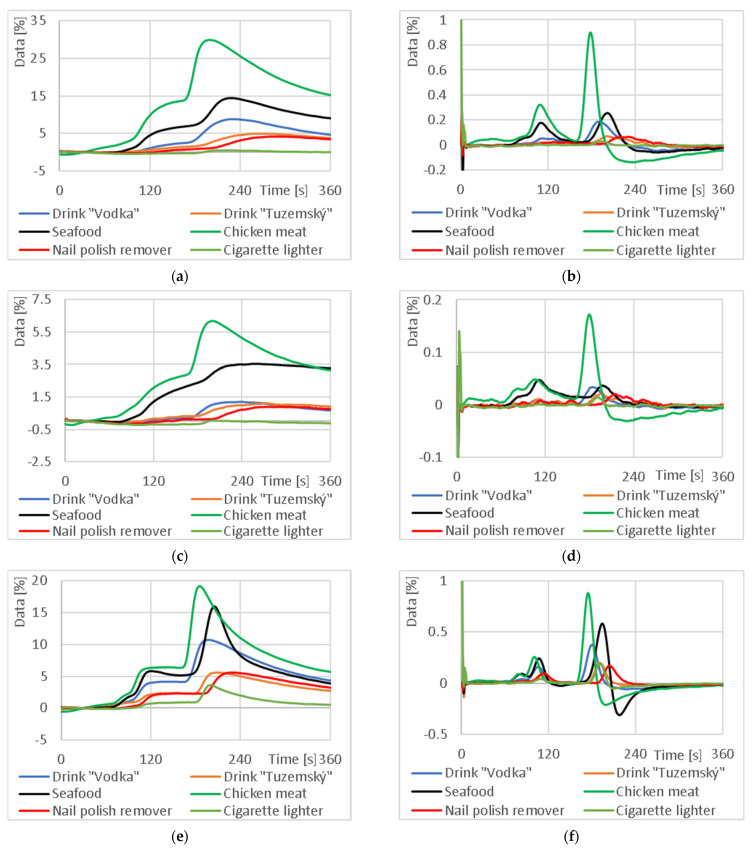
Comparison of output response and absolute increment between test samples using three-tier 3D printed capillaries: (**a**) output response from NH_3_ sensor; (**b**) absolute increment from NH_3_ sensor; (**c**) output response from NO_2_ sensor; (**d**) absolute increment from NO_2_ sensor; (**e**) output response from CO sensor; (**f**) absolute increment from CO sensor.

In the next part, different odor samples were measured and compared, focusing mainly on food. Although the three-tier capillary (3L) had the smallest response at the output of individual sensors in the previous section of all manufactured and tested capillaries, it was chosen for the measurements in this section because of the most and best distributed output signal. These were “Vodka” and “Tuzemský” drinks, seafood, chicken meat, gas from cigarette lighter and nail polish remover. The results are shown in Figure 9.

**Figure 10:**
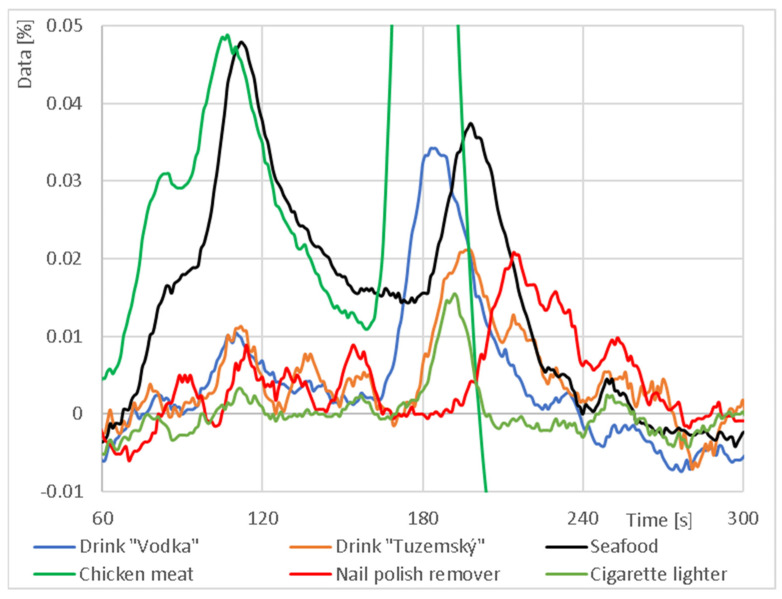
Detail of the absolute signal increment from the NO_2_ sensor for different odor sources.

The results of the output signal comparison indicate that by using this type of 3D printed capillary, it is possible to characterize the odor sample both in terms of magnitude and time. This fact is further confirmed by graphs of absolute signal increments, especially of the chicken meat and seafood signals with the other signals. For example, for the CO sensor, the peak of the maximum decrease for the chicken meat is at 174 s, but, for the nail polish remover, the peak was not observed until 205 s. Furthermore, for the chicken meat and seafood, a signal decrease of around 80 s was detected for the NH_3_ sensor, which was not observed for the other samples. The same characterization of the output signal occurred for the NO_2_ sensor. Here, the signal from the seafood sample had an unusually greater 1st peak drop (109 s) than the subsequent 2nd drop (201 s), while the opposite was true for most of the other samples. A detail of the signal increment is shown in Figure 10.

**Figure 11:**
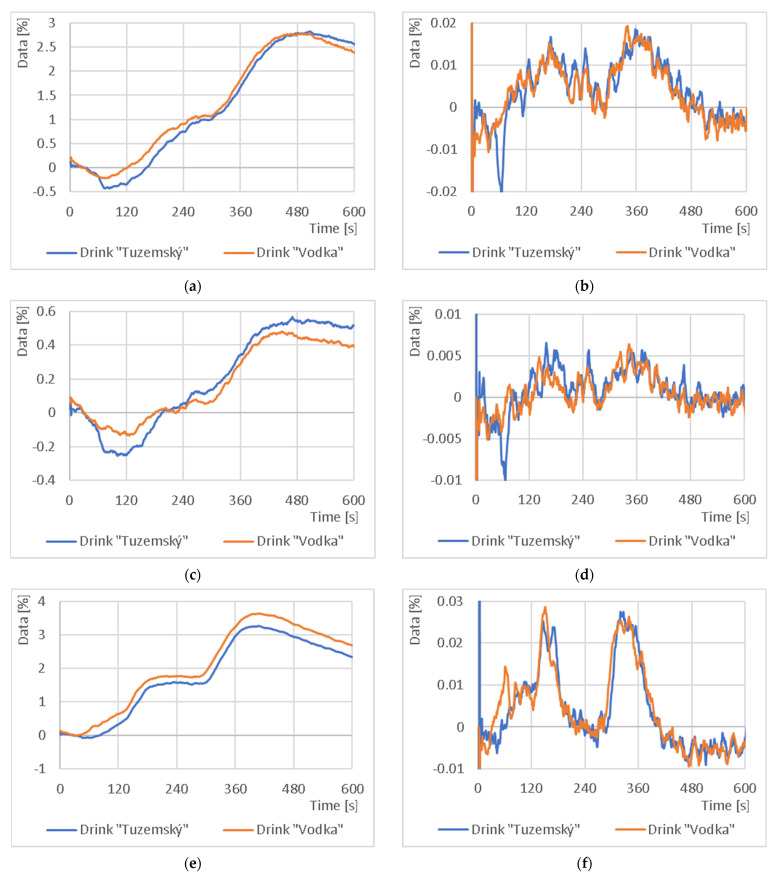
Comparison of output response and absolute increment between test samples using 3-tier 3D printed capillaries: (**a**) output response from NH_3_ sensor; (**b**) absolute increment from NH_3_ sensor; (**c**) output response from NO_2_ sensor; (**d**) absolute increment from NO_2_ sensor; (**e**) output response from CO sensor; (**f**) absolute increment from CO sensor.

In the last part of this study, a comparison of the drink “Vodka” and “Tuzemský” was made at half the carrier gas flow rate. The results are shown in Figure 11. Again, it can be concluded that the mixture was split during the measurement (e.g., the peaks at 150 s in Figure 11f will indicate substance A; the peaks at 330 s will indicate substance B) and the printed capillary added in front of the sensor increased the signal characteristic of the sensor used.

Gas detection is crucial for the food industry, not only in terms of quality control but also safety control. The most commonly detected gases in the food industry are ammonia, ethylene, carbon dioxide and sulfur dioxide; equally important is the detection of volatile substances such as ethanol. These gases can be endogenously produced in food and are thus an important food-quality indicator; they can also be added to food during the production process or during storage and transport. The use of gas sensors for their detection, therefore, plays a crucial role in the food industry [28].

Monitoring of gases such as ammonia or hydrogen sulfide, which are produced by spoilage bacteria, is essential for quality control in products such as meat and seafood [29]. In addition to these gases, a wide range of volatiles can be detected in spoiling meat [30]. Gas sensors have been used successfully to detect meat spoilage before [31], but in this study, individual mixture gases were not detected, and only the sensor response rate was evaluated. Similar results were also achieved by Raudiene et al. [32] by using an electronic nose equipped with three gas sensors (CH, NH_3_ and O_3_ MOS sensors). This study also mentions that the sensors can detect other gases depending on the sensitivity and working range of the sensor used [32]. Benabdellah et al. [33] also suggest that monitoring volatiles such as acetone or ethanol is necessary for early meat spoilage not detectable by the human nose [33]. This supports our suggestion that gases detected in spoilage meat should be pre-separated before being detected by the gas sensor, not only to assess whether the meat is spoiled, but also to potentially characterize the composition of the gas phase using a compact, fast and inexpensive method. For further studies, it would be advisable to validate the results obtained by our work, to extend the range of samples investigated and to complement the experimental setup consisting of a 3D printed column and a MiCS 6814 gas sensor with gas sensors detecting other gases or volatile substances.

Our research group previously published a pilot study combining the use of an electronic nose gas sensor with thermodynamic sensors to obtain broader and more robust results in monitoring the fermentation process of gluten-free flours [34]; therefore, for future experiments, it is also possible to combine multiple sensors and create a device that includes not only a 3D printed column for gas preparative separation but also a multi-sensor system with different detection principles, e.g., for biogenic amines, which are also an indicator of meat spoilage [35]. A fast, inexpensive and accurate assessment of gases from spoiling meat can be a very convenient alternative to the classical methods used in the food industry. One of the most commonly used methods for the determination of ammonia in this field is Nessler’s reagent method. However, this method uses toxic mercury compounds and spectrophotometric determination where fast and accurate work is required [36].

Quick and affordable gas detection also plays a potential role in food sustainability. High losses occur during food transport, according to Gustavsson et al. [37]—up to 1/3 of all food is lost due to inadequate atmospheric monitoring in freight containers. The highest losses occur during transport by cargo ships [37]. A positive impact on sustainability would be not only temperature and humidity control (which are easily monitored) but also oxygen, carbon dioxide and ethylene control. Standard methods for ethylene detection are costly and not very sensitive [38,39].

## 4. Conclusions

In this study, 3D printed capillaries fabricated by the FDM method were designed, fabricated and tested for preparative separation of a gas sample prior to its measurement using a low-cost chemiresistive sensor. A diameter of 1.2 mm was chosen as the optimal capillary diameter because the capillaries collapsed and clogged at smaller diameters. Three types of capillaries with different lengths were designed and fabricated for the preparative separation of the measured gas sample. Clean air was used as the carrier gas. The effect of capillary parameters on the output response of the MiCS6814 sensor was tested on a sample of an alcoholic beverage, “Vodka”. The test results showed successful separation of the output signal into several parts for a three-tier capillary with a length of 4692 mm (the largest designed length) and an internal surface area of 176.96 cm^2^. The successful separation of the output signal into several different parts was further confirmed for other food and non-food samples. Thus, the use of a capillary allowed the output signal from the MiCS-6814 sensor to be specified for a given sample based on signal changes over time.

The pilot study thus demonstrates the feasibility of using a 3D printed capillary fabricated by FDM as a simple, low-cost preparative column for sample gas measurements using chemiresistive sensors and extends the signal evaluation capability of these sensors to the time domain. However, further research using single-component standards is needed to characterize individual signal components more accurately. Further research and testing of the preparative separation capillary design could therefore offer an innovative approach for fast and inexpensive analysis of gas samples in the food industry and other industries, such as, for example, cases of air pollutant detection.

## Figures and Tables

**Table 1:** Capillary length, volume, capillary mass and inner surface depending on their number.

Capillary Tier Number	Tube Lenght [mm]	Tube Volume [mL]	Weight [g]	Inner Surface [cm^2^]
1	1489	1.68	58	56.16
2	3112	3.52	120	117.34
3	4692	5.31	170	176.96

## Data Availability

New research data were presented in this contribution.
